# Will you still need me (Ca^2+^, TnT, and DHPR), will you still cleave me (calpain), when I'm 64?

**DOI:** 10.1111/acel.12560

**Published:** 2016-12-23

**Authors:** José Renato Pinto, Judy Muller‐Delp, P. Bryant Chase

**Affiliations:** ^1^ Department of Biomedical SciencesThe Florida State University College of Medicine1115 West Call StreetTallahasseeFL32306‐4300USA; ^2^ Department of Biological ScienceThe Florida State University81 Chieftain WayTallahasseeFL32306‐4370USA

**Keywords:** troponin T, skeletal muscle, calpain, muscle force, nuclear troponin

Of the many cellular and molecular hallmarks that are broadly associated with physiological decline during aging (López‐Otín *et al*., 2013), loss of muscular strength in vertebrates is particularly problematic because in humans it is a better predictor of morbidity and mortality than loss of muscle mass (Newman *et al*., 2006). Human cohort studies indicate that with both aging and disease, muscular strength is lost more rapidly than muscle mass (Goodpaster *et al*., 2006). The mechanistic changes that underlie age‐related loss of muscular strength, however, have been more elusive to identify than the mechanisms of age‐related sarcopenia. Age‐induced loss of muscular strength has been a topic of sustained debate. Despite a number of plausible hypotheses and clever experimental designs, these earlier studies were unable to dissect the primary mechanism(s) responsible for the reduction in specific force (when the force is normalized to the cross‐sectional area) with aging (Phillips *et al*., 1993; Brooks & Faulkner, 1994; Faulkner *et al*., 2007). More recently, the Delbono group demonstrated that decreased expression of the voltage sensor Ca^2+^ channel α1 subunit (Cav1.1)—also known as the dihydropyridine receptor (DHPR) in the skeletal muscle excitation–contraction coupling literature—is associated with the loss of skeletal muscle strength with aging (Delbono *et al*., 2007; Taylor *et al*., 2009). They also showed that Cav1.1 expression levels can be regulated by different mechanisms, which are not related to gene transcription or mRNA expression (Delbono *et al*., 2007; Taylor *et al*., 2009).

In the paper ‘Calpain inhibition rescues troponin T3 fragmentation, increases Cav1.1, and enhances skeletal muscle force in aging sedentary mice’, Zhang *et al*. report a novel finding that TnT3 regulates Cav1.1 expression in skeletal muscle fibers and that calpain‐mediated fragmentation of TnT3 is associated with Cav1.1 downregulation in old mice (Zhang *et al*., 2016). Delbono and colleagues have described how the age‐induced decrease in Cav1.1 levels leads to uncoupling of the type 1 ryanodine receptor (RyR1), which potentially decreases the amount of activating Ca^2+^ released by the sarcoplasmic reticulum during contraction (Fig. 1) (Delbono, 2011; Hernandez‐Ochoa *et al*., 2015; Lee *et al*., 2015). The coupling between Cav1.1 in the sarcolemma and RyR1 in the sarcoplasmic reticulum membrane has also been recently shown to be modulated by protein Stac3, a novel mechanism for the modulation of excitation–contraction that does not involve changes in the cellular level of Cav1.1 (Polster *et al*., 2016). Importantly, Zhang *et al*. also report that calpain‐mediated TnT3 fragmentation and related downregulation of Cav1.1 expression can be prevented by administration of a calpain inhibitor, BDA‐410, to old mice. These results suggest that calpain activity and reduction in TnT3 fragmentation are potential therapeutic targets for prevention and/or amelioration of age‐related loss of muscular strength.

**Figure 1:**
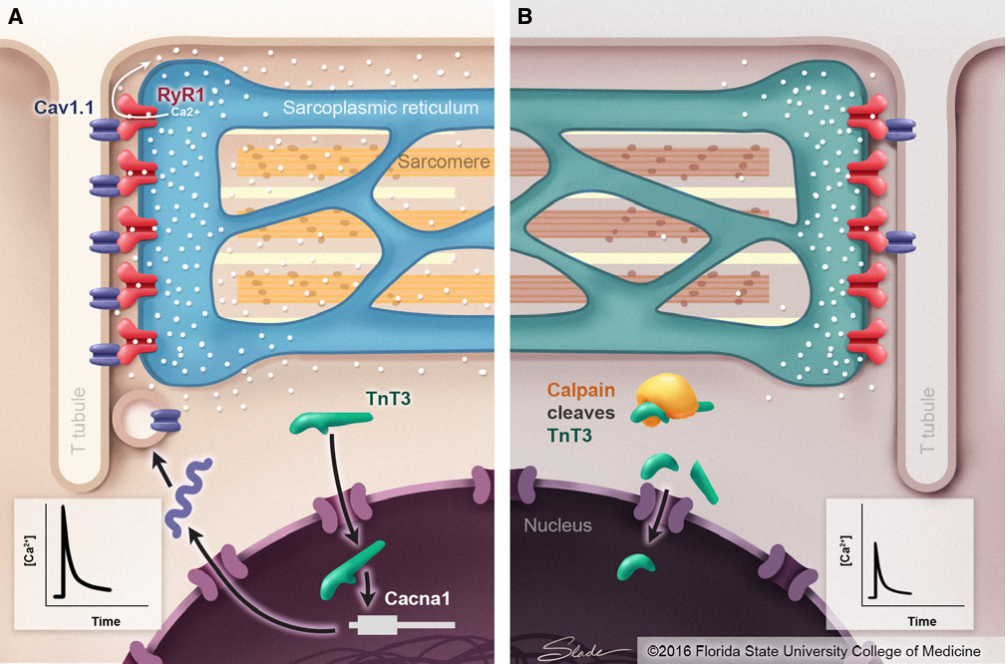
Illustration of the molecular mechanism by which TnT3 regulates expression of Cav1.1 in skeletal muscle. (A) Young skeletal muscle cell and (B) aged skeletal muscle cell.

TnT3 is one of the three polypeptides that comprise the troponin complex in skeletal muscle. The function of the sarcomeric troponin complex in the context of striated muscle regulation has been extensively studied (Farah & Reinach, 1995; Gordon *et al*., 2000; Vinogradova *et al*., 2005). In addition to cytoplasmic localization in thin filaments of the sarcomere, TnT and troponin I (TnI)—along with troponin C (TnC), tropomyosin, and actin—are present in the nuclei of striated muscle cells (Asumda & Chase, 2012; Chase *et al*., 2013; Zhang *et al*., 2013a,b). However, the role of troponin subunits in the striated muscle nucleus is still under investigation and little is known. A mutant human cardiac TnT (cTnT‐R173W) associated with dilated cardiomyopathy (DCM) accumulates in the nuclei of iPSC‐derived cardiomyocytes and upregulates phosphodiesterase (PDE) 2A and 3A activities via modulation of epigenetic factors (Wu *et al*., 2015). Increased PDE activity is correlated with increased cAMP levels and impaired β‐adrenergic signaling, which are hallmarks of the disease in patients with DCM and which compound the detrimental effects of the TnT mutation on sarcomere contractility. In the paper by Zhang *et al*. (2016), TnT3—the TnT isoform found in fast‐twitch skeletal muscle fibers—is reported to bind to the promoter region of the *Cacna1s* (gene encoding Cav1.1) and regulate its transcription levels in skeletal muscle fibers.

In Zhang *et al*., the authors demonstrated that the expression of Cav1.1 is coupled to the expression of TnT3 and that TnT3—but not TnI, TnC, or tropomyosin—binds specifically to the promoter region of *Cacna1s*. Then they used SDS‐PAGE to separate nuclear protein extracts obtained from old mice (23–25 months) and identified a fragment of TnT3. Mass spectrometry analysis indicated that cleavage sites of the TnT3 fragments corresponded to sequences targeted by calpain. Subsequently, the authors elegantly showed that old mice treated with BDA‐410 (a synthetic Leu–Leu peptidomimetic that inhibits cysteine proteases) daily for 21 days displayed increased absolute and specific forces at all frequencies of stimulation tested in intact soleus muscle preparations, without changes in CSA. The endurance capacity tested *in vivo* and *ex vivo* was not improved by the BDA‐410 treatment. Furthermore, they showed that BDA‐410 does not affect the composition of soleus and EDL muscles; for example, the levels of titin and myosin heavy chain (MHC) were not altered as well as MHC isoform distribution in these two muscles. Interestingly, BDA‐410 treatment stabilized the nuclear full‐length TnT3 and decreased the levels of fragmented TnT3, which consequently increased the amount of Cav1.1 in skeletal muscle cells (Fig. 1) (Zhang *et al*., 2016).

Current therapeutic approaches that are being tested to improve skeletal muscle function in the context of aging and disease include myostatin inhibition, hormone therapy, and troponin activation, among others (Jasuja & Lebrasseur, 2014). Calpain activity has been linked to several disease conditions in striated muscle (Jia *et al*., 2001; Witt *et al*., 2004; Zhang *et al*., 2006; Patterson *et al*., 2011), and inhibition of its activity has been suggested as a therapeutic strategy (Carragher, 2006), leading to the identification of BDA‐410—the calpain inhibitor used by Zhang *et al*.—and the search for other small molecule inhibitors (Xu *et al*., 2013). The report by Zhang *et al*. demonstrates a role for nuclear TnT3 regulating *Cacna1s* transcription and Cav1.1 expression, and it also indicates that inhibition of skeletal muscle calpain activity may be an effective new therapeutic strategy to diminish the deleterious effects of aging on muscle function. Another potential topic of investigation is whether resistance training, which is known to effectively increase strength through mechanisms other than hypertrophy in aged muscle (Frontera *et al*., 1988), exerts its beneficial effect on muscle performance by decreasing nuclear fragmented TnT3. This study by Zhang *et al*. provides both important mechanistic data for understanding the regulation of Cav1.1 in aged skeletal muscle and direction for future interventional studies in the aging population.

## Funding

National Heart, Lung and Blood Institute of the National Institutes of Health Grant HL128683 to JRP.

## Conflict of interest

None declared.
